# Understanding Seepage in Levees and Exploring the Applicability of Using an Optical-Fiber Distributed Temperature System and Smoothing Technique as a Monitoring Method

**DOI:** 10.3390/s23104780

**Published:** 2023-05-16

**Authors:** Woochul Kang

**Affiliations:** Department of Hydro Science and Engineering Research, Korea Institute of Civil Engineering and Building Technology, Goyang 10285, Republic of Korea; kang@kict.re.kr; Tel.: +82-10-8505-0471

**Keywords:** distributed temperature system, seepage, levee, smoothing, optical fiber

## Abstract

This study aimed to experimentally understand the seepage mechanism in levees and evaluate the applicability of an optical-fiber distributed temperature system based on Raman-scattered light as a levee stability monitoring method. To this end, a concrete box capable of accommodating two levees was built, and experiments were conducted by supplying water evenly to both levees through a system equipped with a butterfly valve. Water-level and water-pressure changes were monitored every minute using 14 pressure sensors, while temperature changes were monitored using distributed optical-fiber cables. Levee 1, composed of thicker particles, experienced a faster water pressure change, and a corresponding temperature change was observed due to seepage. While the temperature change inside the levees was relatively smaller than external temperature changes, measurement fluctuations were significant. Additionally, the influence of external temperature and the dependence of temperature measurements on the levee position made intuitive interpretation challenging. Therefore, five smoothing techniques with different time intervals were examined and compared to determine their effectiveness in reducing outliers, elucidating temperature change trends and enabling the comparison of temperature changes at different positions. Overall, this study confirmed that the optical-fiber distributed temperature system combined with appropriate data-processing techniques can be more efficient than existing methods for understanding and monitoring levee seepage.

## 1. Introduction

Owing to recent climate changes, the frequency and magnitude of abnormal floods have increased, thereby highlighting the need to secure and maintain the stability of levees that protect human life and properties from river floods. The primary causes of levee failure include overflow, seepage, embankment instability, and failure of river structures [1,2,3]. Seepage in levees can result from embankment leakage caused by poor filling materials and compaction or ground leakage due to faulty foundation treatment. Differential head between inland and riverside land causes seepage in levees, leading to an expansion of the seepage face, an increase in pore water pressure, and a reduction in the shear strength of the soil. Piping can occur when streamlines are concentrated due to seepage, resulting in the collapse of the levee. To accurately understand and analyze the seepage mechanism in levees, the factors affecting it, and its importance in levee stability, several studies have been conducted, involving experiments, field investigations, and models. Factors such as the geometric characteristics, geological factors (slope, size, and degree of compaction), soil permeability, grain distribution, and homogeneity of hydraulic conductivity influence levee failure to various degrees [4,5]. In particular, the mechanism behind levee failure caused by seepage is as follows. Seepage causes a decrease in soil particle resistance, leading to the initiation of internal erosion, i.e., the removal of soil particles by the flowing water, which, in turn, marks the beginning of the piping process [6]. As leakage progresses through the generated gap, flow occurs under the levee or through the levee strata, causing further internal erosion and a reduction in soil stability, ultimately weakening the levee structure [7,8]. Furthermore, when a levee is exposed to high water levels over an extended period, it can become saturated, leading to failure [9].

Since monitoring the seepage process is crucial in preventing levee failure, extensive research has been carried out to develop efficient monitoring systems [10]. Conventional seepage monitoring methods include visual inspection to identify areas susceptible to collapse and using geotechnical equipment based on pore water pressure [11,12]. However, visual inspection is an inefficient method as it requires experts to perform regular inspections, whereas detecting local effects using equipment that relies solely on pore water pressure is very challenging [10]. Furthermore, the continuous monitoring of seepage in a levee necessitates information on time and space, which is difficult to secure using conventional methods [13]. Additionally, methods based on electrical resistances or magnetoelectric measurements can only analyze indirect effects related to erosion inside levees [11]. To address these shortcomings, the temperature-based monitoring method was proposed in the 1950s as an efficient levee monitoring method [13]. Since the 1980s, studies have been conducted for monitoring seepage using distributed temperature sensors (DTS) [13,14,15]. The DTS-based method is very efficient compared to the traditional methods because it can overcome spatial limitations, and these methods can be classified into two types: passive and active [11]. The studies related to DTS-based method have focused on improving methods that measure seepage in levees and generate appropriate alarms, which has resulted in the development of active methods that measure the temperature under the application of heat to identify seepage characteristics [13]. On the other hand, understanding the changing correlation between soil moisture and temperature fluctuations over time is crucial for the successful application of DTS technology in the passive method, which directly measures the temperature inside the embankment [16]. When using the passive method that measures the natural temperature of the levee, it is necessary to conduct seepage analysis considering various factors, such as air temperature, soil temperature, and groundwater. Additionally, the temperature inside a levee is influenced by the upstream reservoir and abnormal flow [17]. It means that applying data processing is required to obtain accurate temperature information related to seepage [13]. 

This study aims to investigate seepage in levees by conducting a real-scale experiment. Two levees with a height of 2 m, a slope of 1:2, and a width of 5.5 m were constructed in a concrete box to minimize the scale effect and control various factors affecting seepage in levees. In this experiment, distributed optical-fiber cables were used to monitor the temperature inside the levee via a passive method, and the applicability of DTS was tested by measuring water pressure with a pressure sensor. The temperature data obtained through this method exhibit propagation uncertainty due to severe fluctuations and considerable noise, necessitating appropriate post-processing. Therefore, various smoothing methods were applied to determine the practical utility of the temperature data obtained from the DTS. Finally, the smoothed results were analyzed to gain insight into the seepage process and mechanism in levees. 

## 2. Experiment and Analysis Methods

### 2.1. Experimental Conditions and Methods

**Figure 1:**
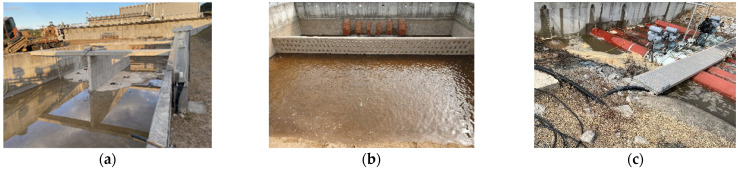
(**a**) Concrete box fabricated for the real-scale levee seepage experiment, (**b**) flow supply and control system, and (**c**) perforated concrete to maintain water level.

One of the primary purposes of the experiments in this study is to understand the seepage mechanism based on the natural temperature inside real-scale levees measured using distributed optical-fiber cables. First, a concrete box was fabricated to control various external factors that affect the seepage process of levees (presence/absence of groundwater and change in seepage depending on levee foundation conditions) and to examine the seepage process under the same environmental conditions (Figure 1a). 

**Figure 2:**
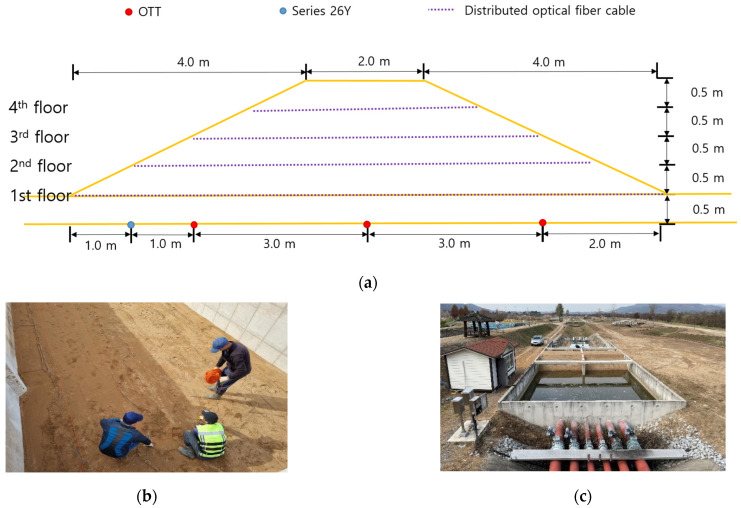
(**a**) Schematic of a real-scale levee for the seepage experiment, (**b**) levee construction process and optical cable installation, and (**c**) constructed levee and experiment in execution.

In the middle of the concrete box, a concrete wall was prepared so that two levees could be constructed to compare the experimental results (Figure 1a). In addition, on one side of the fabricated concrete box, water was supplied using a tubular flow supply system controlled by a butterfly valve, and a distributing plate was installed to supply water evenly throughout the box and in a stable manner (Figure 1b,c). Six holes were drilled at the top of the wall on the water supply side to maintain a constant water level from the continuously supplied flow (Figure 1c). After separately creating a foundation layer 0.5 m thick, considering the seepage flow caused at the interface between the concrete of the box and the soil of the levee, two levees with a height of 2 m, length of 10 m, width of 5.5 m, and slope of 1:2 were constructed (Figure 2a). 

**Figure 3:**
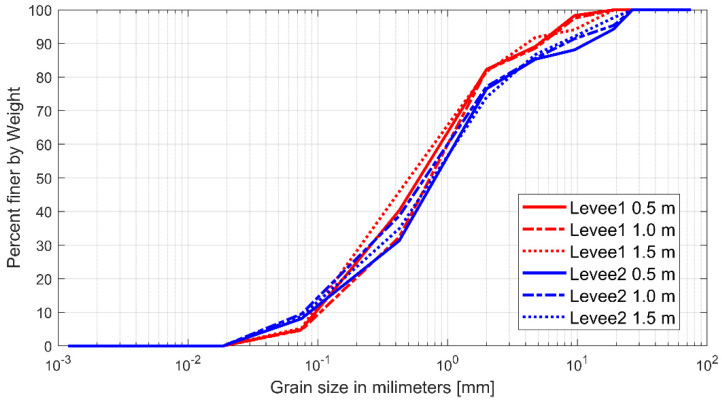
Particle size distribution curve of the soil used for the embankment of the two levees.

During the levee construction process, the basic embankment foundation was created by dropping sand from 4 m to 5 m using an excavator (0.2 m^3^). Subsequently, levees were piled up at 0.5 m high intervals using a mini excavator, and back-and-forth compaction was performed once using a 1-T compacting roller to make the levees solid. In addition, considering that the seepage process could be different between the two levees if the degree of compaction was not uniform, the two levees were compacted alternately every 0.5 m in the same way to derive stable experimental results. To satisfy the geological condition of constructing the two levees with sand having the most uniform particle size distribution, which is one of the conditions affecting seepage, the sand collected through sifting for selecting sand particles with a size of less than 2 cm was used. To examine whether the levees were made of uniform sand, a particle size analysis was conducted on the sand that constituted the embankment at 0.5, 1.0, and 1.5 m, except for the foundation of both levees (Figure 3).

For levee 1 (right levee in Figure 1c), the material was classified as well-graded sand (SW) based on the unified soil classification system, and the moisture contents of the soil samples collected before the experiments at heights of 0.5, 1, and 1.5 m were 3.7%, 4.3%, and 3.9%, respectively. In the case of levee 2, some materials were classified as sand mixed with silt (SW-SM), and the moisture contents at the same heights were 4.8%, 8.6%, and 5.6%, respectively. Finally, the particle size distribution confirmed that levee 1 was composed of particles thicker than that of levee 2. 

### 2.2. Measurement Method

**Figure 4:**
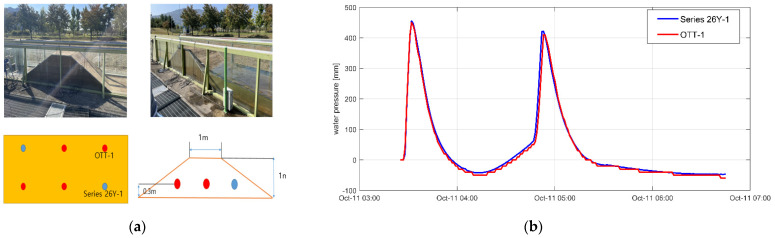
(**a**) Preliminary experiment for calibrating sensors and (**b**) pressure-change measurement results.

Water pressure was measured to confirm the seepage phenomenon. For this, a piezometer is generally used. This study measured the change in water pressure by installing two digital pressure sensors (KELLER Pressure Series 26Y, Series 26Y) connected to a data logger (DT 80) and 12 pressure-type water gauges with a built-in pressure sensor (OTT Orpheus mini water level logger, OTT) on the concrete ground under the levee foundation layer. Series 26Y can measure from 0 m to 100 m with a pressure measurement accuracy of ±0.25% FS. In the case of the OTT, the accuracy of the pressure sensor is ±0.05% FS in the (0 to 100) m range. In addition, the resolution of the OTT is 0.01% FS and that of Series 26Y is 0.001% FS. In the case of OTT, the water pressure can be measured with a built-in pressure sensor, but a preliminary experiment was performed by fabricating a small levee because its measurement range and resolution performance can be low (Figure 4).

**Figure 5:**
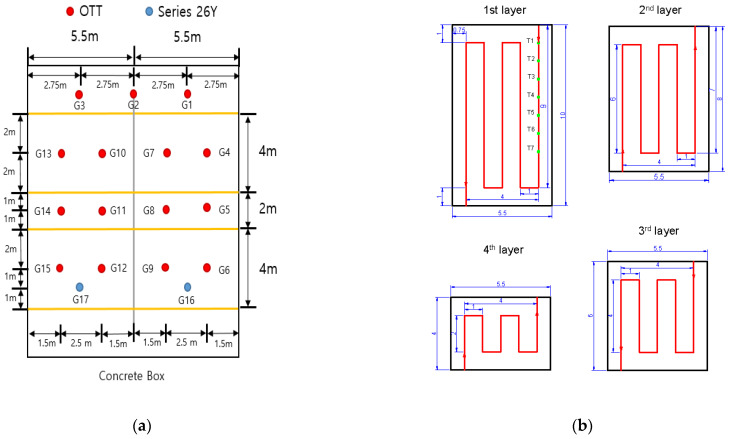
Positions of the (**a**) pressure sensors and (**b**) distributed optical-fiber cables on each layer used for the experiment (All figures are presented in flow direction and dimensions in meters).

As this experiment was performed to examine the differences in sensor readings, a random water level condition was maintained. The experimental results confirmed that the pressure change caused by seepage can be analyzed using these sensors although there were slight errors depending on the sensor position and resolution. Therefore, in the seepage experiments conducted on the real-scale levees, two sensors were installed on the concrete ground (Figure 5) and operated simultaneously, and the pressure change caused by seepage was measured every second. In addition, distributed optical-fiber cables were installed to understand the seepage behavior in the levees and examine their potential in monitoring the changes, as shown in Figure 2b and Figure 5. 

For the distributed optical-fiber cables, when a laser pulse was incident, the reflected wave changed with changes in the surrounding environment, and the type of scattering could be distinguished by the changed wavelength. Note that Raman-scattered light is favorable for increasing the temperature measurement resolution owing to its large wavelength shift caused by the change in ambient temperature [18,19]. However, this study used the passive method, which is highly economical compared to the active method primarily used in seepage experiments. In addition, the temperature changes inside the levees caused by simple seepage were measured using optical fiber sensors while controlling various factors that affect the temperature change and seepage process. The optical fiber used in the experiment had a multimode-graded index with a core diameter of 50 μm and a cladding of 125 μm, primarily used for temperature measurements. The fiber optic cable was composed of SUS304 (stainless steel), yarn, braid, and a low-smoke zero-halogen jacket to physically protect it from external factors. Temperature was measured every 14 s at 1 m length intervals. The fiber cables used can measure temperatures ranging from −20 °C to 95 °C, with an accuracy of approximately 1 °C and a spatial resolution of less than 1 m. 

To understand the levee seepage mechanism from the measured temperature data, the smoothing process, which is a method for simplifying data by reducing noise or variability while preserving the underlining features of the data, was applied. This study initially determined an appropriate time range for smoothing the temperature data inside the levees. Subsequently, the following five smoothing methods were applied: (1) the moving-average method, (2) the Savitzky–Golay method, (3) the locally estimated scatterplot smoothing (LOESS), (4) the locally weighted scatterplot smoothing (LOWESS), and (5) the exponential smoothing method. The moving-average method smooths the data by replacing each data point with the average of its neighboring data points (Equation (1)).

(1) $$y_{s}\left(i\right)=\frac{1}{2N+1}\left(y\left(i+N\right)+y\left(i+N-1\right)+\cdots +y\left(i-N\right)\right)$$

where $y_{s}\left(i\right)$ is the smoothed result of the i-th data, N is the number of neighboring data points on both sides, and 2N+1 is the span. This method is effective in reducing noise and outliers while preserving the features of the data. The Savitzky–Golay method is a generalized moving average method. The filter coefficients are obtained by fitting an unweighted linear least squares polynomial of a specified degree. It is frequently applied to frequency or spectral data because it preserves high-frequency components through polynomial approximation [20,21]. The LOWESS and LOESS methods smooth the data based on locally weighted linear regression (Equation (2)).

(2) $$wi={\left(1-{\left|\frac{x-x_{i}}{d\left(x\right)}\right|}^{3}\right)}^{3}$$

where wi is the weight, ***x*** is the predictor value to be smoothed, $x_{i}$ is the nearest neighbor of ***x*** within the search range, and ***d***(***x***) is the distance to the farthest predictor value from ***x*** within the range along the abscissa. These are non-parametric regression methods frequently used in time series data analysis to fit locally weighted regression to a scatter plot of data [22]. This method can change the characteristics of the time series data by resizing the LOESS smoothing window and can estimate trends more accurately than other smoothing methods. The two methods have a significant difference in weight function. In detail, the LOWESS method has low computational cost because it does not need to solve an optimization problem. Finally, the exponential smoothing method assigns exponentially decreasing weights to past observations and the highest weight to the most recent observation. This method is also generally used for time series data; however, it may not be suitable for data with specific seasonality patterns and may not work well for noisy data [23,24]. 

## 3. Results

### 3.1. Experimental Results

**Figure 6:**
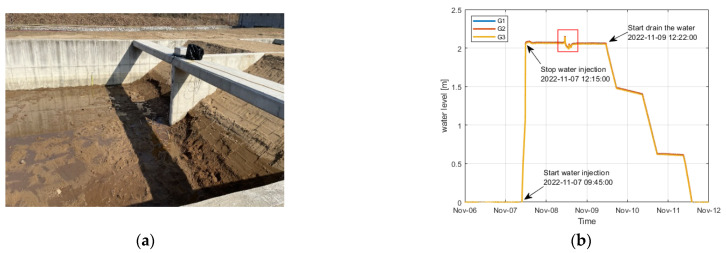
(**a**) Final levee condition with no change in external seepage line and (**b**) water level change measurement results.

The seepage experiment in the real-scale levees was performed from 7 to 13 November 2022. To examine the change in water pressure and temperature caused by seepage, water was slowly supplied from 09:45 a.m. to 12:15 p.m. on November 7 until a water level of approximately 80% of the levees (2.1 m) was reached (Figure 6a). From 12:22 p.m. on November 9, when it was confirmed that the levees were fully saturated and there was no more change in the seepage line outside the levees, the filled water began to be drained (Figure 6b). 

**Figure 7:**
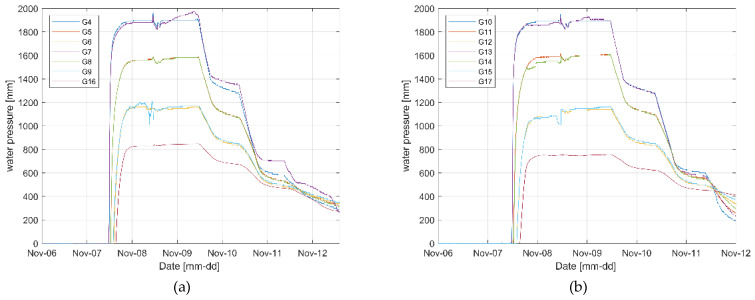
Water pressure change results from seepage (**a**) levee 1 and (**b**) levee 2.

Figure 7 shows the water pressure change results measured from each sensor after water injection into the concrete box to create seepage conditions. 

The two levees showed similar water-pressure change results, but the water pressure of levee 1 was relatively higher at some positions than levee 2. This appears to be because of the difference in the particle size of soil that constituted the embankment and the degrees of compaction that were not perfectly identical, considering that the water level was constant (Figure 6b) and other external environmental conditions that affect seepage were set to be as constant as possible. In the case of levee 1, the water pressure at G16 was relatively higher than that at G17 in levee 2. This is because the embankment was constructed using soil with a larger particle size. Table 1 presents the results of comparing the time when the water pressure began to increase and the time when the section with little change in water pressure began from the sensors at each position between the two levees. In the case of levee 1, the time for flattening after the change in water pressure reached the high water-level condition was generally shorter than that of levee 2. This is because of the difference in the particle size, as observed in the above results. In addition, the water-pressure change reaction was faster in the section where the high water-level condition was artificially changed (Figure 6b red box). This is because the embankment was already saturated. In the case of some water pressure measurement results (G7, G8, G9, G13, and G15), irregular water pressure changes occurred, indicating that seepage intensively occurred in the seepage water flow paths generated from the difference in the compaction degree and particle size in the case of seepage in the embankment. 

### 3.2. Temperature

**Figure 8:**
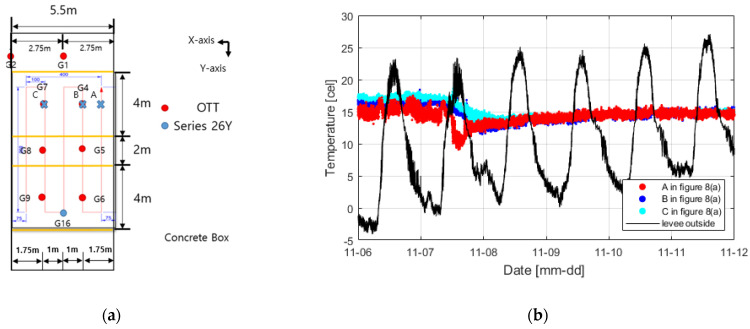
(**a**) Sensor positions in the levee and (**b**) temperature change results inside and outside the levee owing to seepage.

Figure 8a shows the results of measuring the temperature change according to the water-pressure change using the installed distributed optical-fiber cables. 

**Figure 9:**
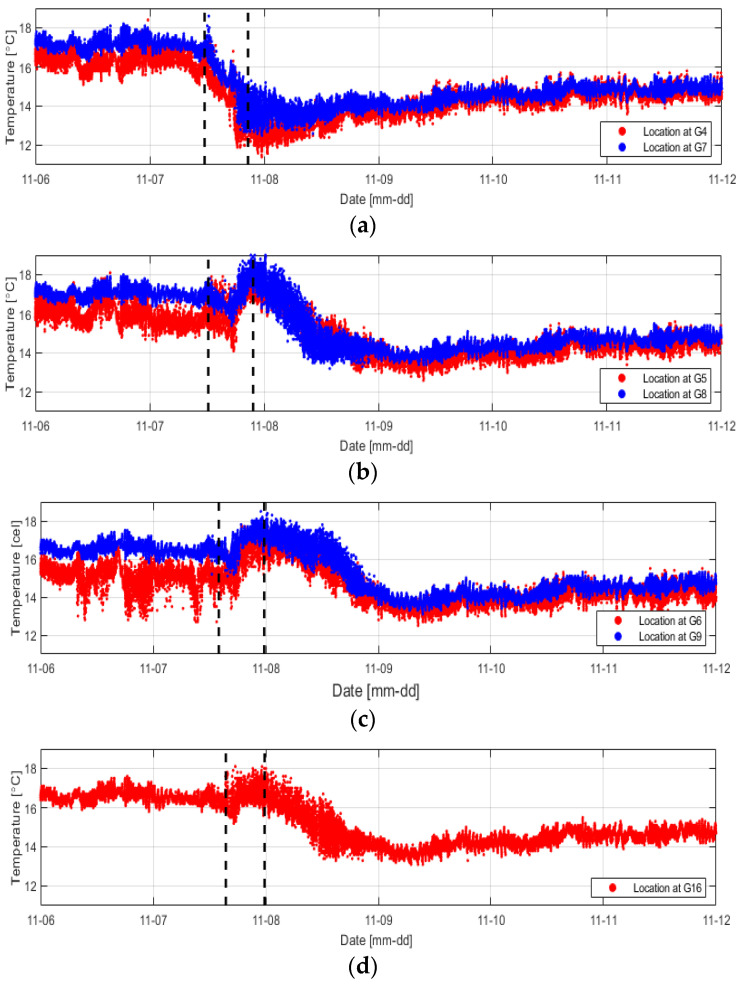
Temperature change at sections where the water pressure was changed by seepage (**a**) 2 m, (**b**) 5 m, (**c**) 8 m, and (**d**) 9 m.

The temperature change inside the levee was negligible compared to the external temperature, but the temperature fluctuation was severe in a small range. Measurements on November 6 before the water supply confirmed that the internal temperature slightly increased as the external temperature increased and that the temperature inside the levee decreased since when water was supplied. Figure 9 shows the temperature change with time compared in Table 1 at various water-pressure-change measurement positions (G4, G5, G6, G7, G8, and G9).

At positions close to water (G4 and G7), the temperature change occurred instantly as they were rapidly affected by seepage owing to the low levee height and proximity to water. At the other positions, however, the temperature was decreased by seepage and then increased again under the influence of the external temperature rise. Subsequently, the temperature continuously decreased while the levee was completely saturated. In conclusion, regarding temperature change, it is possible to monitor the time of seepage and sections where the seepage occurs more rapidly, but it is challenging to confirm the stage at which seepage begins because the temperature change is small and is still affected by the external temperature. It was possible, however, to confirm the reduction in temperature due to the saturation by seepage. In addition, as the temperature is different at each position, it is necessary to closely examine the temperature data inside the levee in terms of time and space.

### 3.3. Data Processing

**Figure 10:**
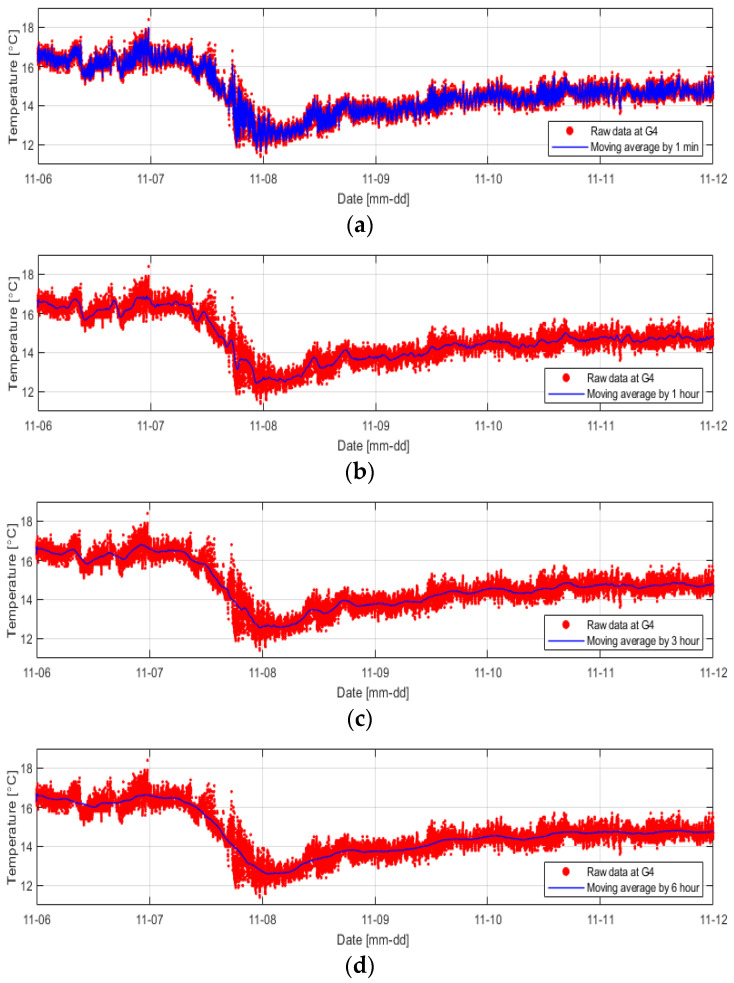
Results of smoothing the temperature data obtained from the G4 sensor position using the moving average method at intervals of (**a**) 1 min, (**b**) 1 h, (**c**) 3 h, and (**d**) 6 h.

The temperature data inside the levee obtained using distributed optical-fiber cables had a narrow range but exhibited severe fluctuations. Therefore, an appropriate data processing method is required to understand temperature data more intuitively. In this study, the temperature data were smoothed at 1-min, 1-h, 3-h, and 6-h intervals, respectively, using the most used moving-average smoothing method (Figure 10). 

**Figure 11:**
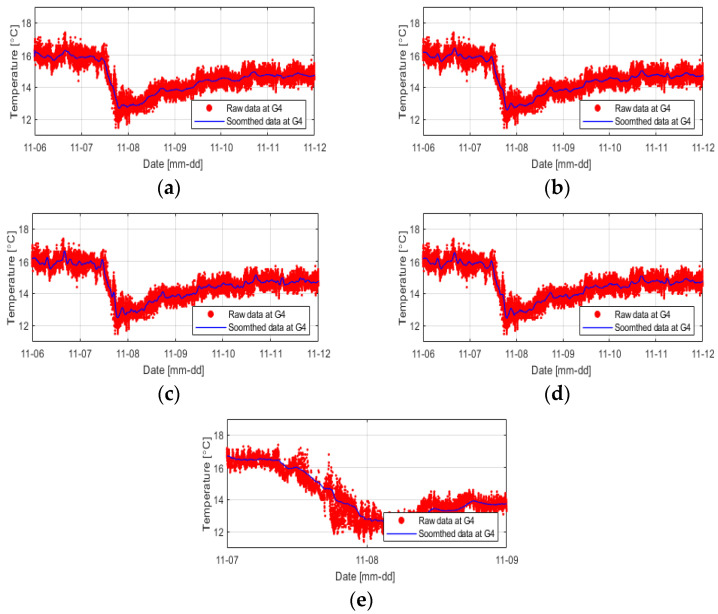
Results of smoothing the temperature data obtained from the G4 sensor position at 3 h intervals using (**a**) moving average, (**b**) first-order local linear regression, (**c**) second-order local linear regression, (**d**) Savitzky–Golay, and (**e**) exponential smoothing methods.

As expected, the data smoothed at 1 min intervals could not effectively reduce severe temperature fluctuations, while the data smoothed at 6 h intervals presented too simplified results. In this study, smoothing was performed using five different methods at 3 h intervals under the judgment that smoothing the temperature change at 3 h intervals well reflected the temperature change tendency, and it would make it easy to compare with the temperature data at other positions (Figure 11). 

**Figure 12:**
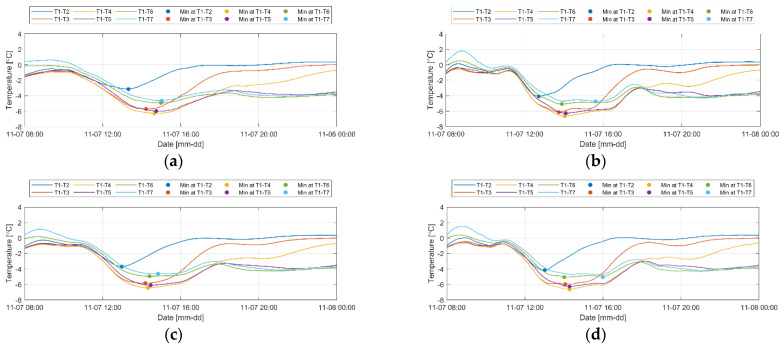

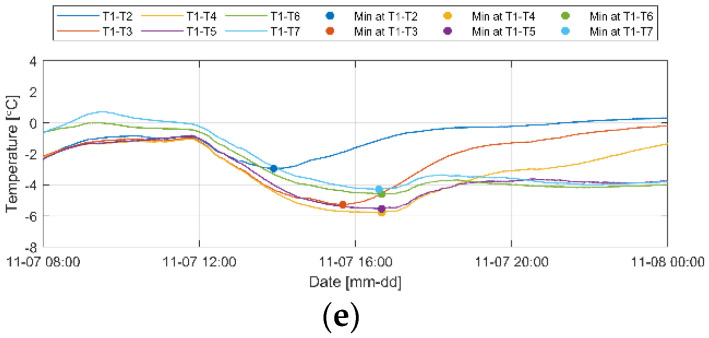
Results of smoothing the temperature difference values obtained from the distributed optical-fiber cables installed on the rightmost side of the foundation layer of levee 1 using (**a**) moving average, (**b**) first-order local linear regression, (**c**) second-order local linear regression, (**d**) Savitzky–Golay, and (**e**) exponential smoothing methods (refer to Figure 5b for the locations of points T1~T7).

The Savitzky–Golay method, which shows benefits for data processing in the high-frequency region where data fluctuations occur rapidly, and the LOWESS smoothing method, which is useful when nonlinearity or outliers are included, were used for processing the temperature data, and more detailed temperature changes were observed than that observed using the other methods. Exponential smoothing method, which assigns exponentially decreasing weights to past observations, provided excessively smoothed results. To compare each smoothing method, the differences in the temperature values over time obtained from the rightmost columns (T1 to T7) in the levee foundation layer were compared (Figure 12). 

In the case of levee seepage, since the occurrence of seepage is further delayed as the distance from the water-filled area (the *y* value) increases, the appearance of the minimum value of the temperature difference is also delayed as the distance increases. Taking this into account, the results of the moving average smoothing method (Figure 12a) could be judged as the most reasonable in this study, which could be attributed to the absence of seasonality or periodic variability in the temperature change resulting from seepage, and its excellent ability to remove outliers by relying only on previous data. However, it was confirmed that the minimum temperature value itself has no meaningful interpretation due to the spatial difference caused by external factors that affect the temperature change. 

## 4. Discussion

**Figure 13:**
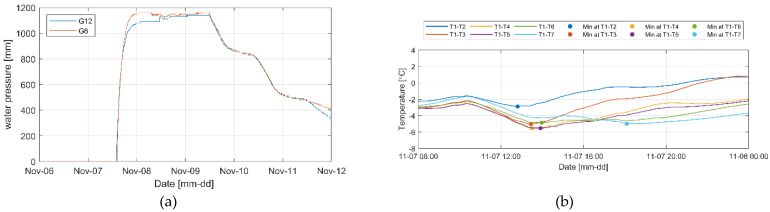
Results of smoothing (**a**) the water pressure difference at 8 m positions for both levees and (**b**) the temperature difference values obtained from the distributed optical-fiber cables installed on the rightmost side of the foundation layer of levee 2 using the moving average method.

To examine the temperature change and seepage mechanism caused by seepage, an analysis was conducted similar to that for levee 2 (Figure 13).

**Figure 14:**
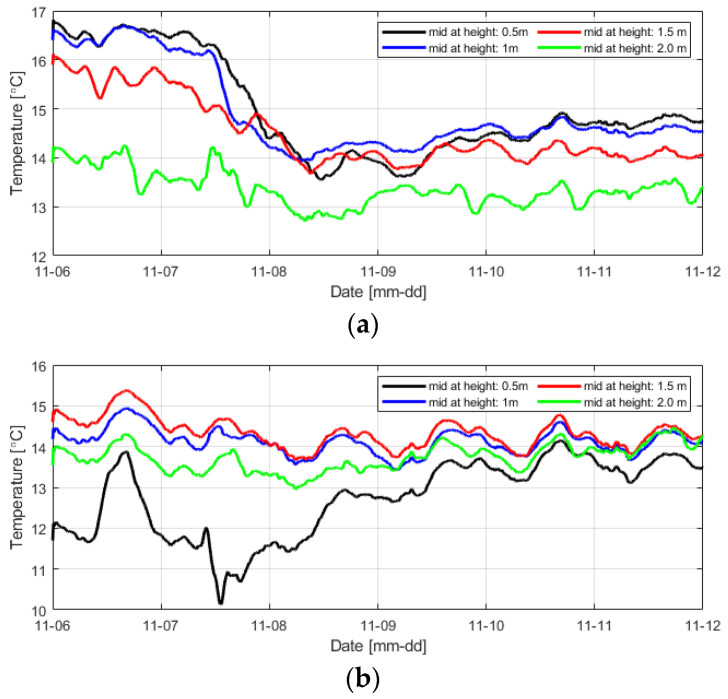
Temperature change per hour due to seepage at different heights in the middle of the levee (x = 3 m and y = 5 m) for (**a**) levee 1 and (**b**) levee 2.

The temperature difference results for levee 2 at the same positions as those analyzed in levee 1 indicated that the minimum value also appeared late as the distance from the water-filled area increased. For the difference between T1 and T7, however, the minimum value appeared considerably late. This result indicates that less seepage occurred and more time was required for saturation in levee 2 compared to levee 1 based on the finding that the water pressure of G17 was low. Figure 14 shows the smoothed temperature change results at different heights in the middle of the two levees. 

**Figure 15:**
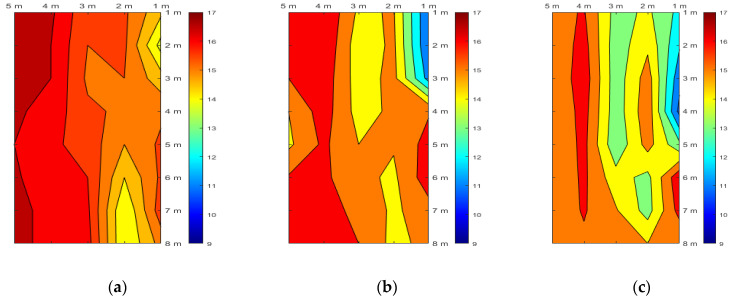

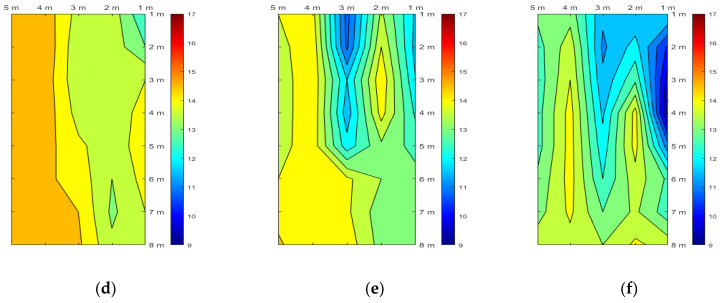
Temperature change caused by seepage over time across the foundation layer (at a height of 0 m) in levee 1 on (**a**) 11-07 09:30, (**b**) 11-07 12:30, and (**c**) 11-08 00:00 and in levee 2 on (**d**) 11-07 09:30, (**e**) 11-07 12:30, and (**f**) 11-07 18:30.

As the water pressure measured after the water supply was approximately 1.6 m near the position where the comparison was made, the positions of the two levees at a height of 2 m showed similar initial temperature change tendencies as they were significantly affected by the external temperature. However, the temperature was low, and its change was small for levee 2 until water was supplied because its soil moisture content was relatively high. After the water supply to examine the seepage effect, levee 1 showed similar temperature change trends except for the top layer. In the case of levee 2, the temperature rapidly decreased after seepage only in the bottom layer, and similar temperature changes were observed in the other layers. Consequently, it appears that seepage progressed in all layers for levee 1. In the case of levee 2, it is judged that seepage in the form of piping or foundation leakage primarily occurred in the bottom layer containing many coarse particles. In addition, only for the section where the temperature sensor was installed in the foundation layer (levee height = 0 m), the temperature changes of the two levees over time are shown in Figure 15. 

As the water pressure in the embankment increased in the foundation layer of levee 1 due to seepage, it can be intuitively confirmed that the overall temperature in the embankment decreased. A similar temperature change trend was also observed in levee 2. For both levees, the temperature change at positions close to the right concrete wall (X-axis = 1 m) were faster than that at other positions. A similar tendency was also observed in the water-pressure measurement results as the water pressures at G4, G10, and G11 located on the right side were higher than those at G7, G13, and G14 in the same period. While the average temperature decreased from 15.8 °C at 09:30 to 15.1 °C at 16:30 on 7 November for levee 1, it decreased from 14.1 °C to 12.7 °C during the same period for levee 2. This result also confirms that levee 2 was subjected to more intensive seepage in the foundation and bottom layers. 

## 5. Conclusions

This study conducted real-scale levee experiments to investigate the seepage mechanism in river levees and evaluate the potential of distributed temperature sensors for monitoring levee stability. To control external factors that affect seepage, a concrete box capable of accommodating two levees was constructed. A distributing plate was installed to supply water evenly to both levees through a flow-supply system equipped with a butterfly valve. A 0.5 m high layer of sand was filled in a concrete box as the foundation layer, and the two levees with a height of 2 m, length of 10 m, width of 5.5 m, and slope of 1:2 were constructed using the same compaction process. Although the characteristics and particle size of the soil used to construct the levees were kept as uniform as possible, the sand particles in levee 1 were thicker, whereas levee 2 contained more silt. 

Two pressure sensors (Series 26Y) and 12 pressure-type water gauges with a built-in pressure sensor (OTT) were installed on the concrete ground to measure the water-pressure change per minute inside the levees caused by seepage. Additionally, distributed optical-fiber cables that use Raman-scattered light were installed inside the levees at heights of 0, 0.5, 1, and 1.5 m to record the temperature change inside them every 14 s at 1 m intervals. Water was slowly supplied for 3 h to induce seepage in the levees. Subsequently, a water level of 2.1 m was maintained for approximately 48 h, and water pressure was measured at 14 positions. The results show that similar water-pressure changes occurred in both levees after the start of seepage, but the water-pressure change was relatively fast, and high water pressure was recorded by some pressure sensors in levee 1 owing to the difference in particle size. The temperature change inside the levees, as measured by the optical-fiber distributed temperature system, was negligible compared to the external temperature, but severe fluctuations were observed. Saturation instantly occurred at positions directly affected by seepage, resulting in a decrease in temperature inside the levees. However, intuitive interpretation became difficult as the distance increased owing to the influence of the external temperature at different positions. Therefore, an analysis was conducted to understand the levee seepage mechanism by comparing and examining five smoothing techniques and appropriate time intervals for their application. In this study, 3 h intervals were selected as the optimal interval because it adequately reflected the temperature change tendency and made it easier to compare the temperature changes measured at different positions. By comparing the temperature differences at seven distributed optical-fiber cable positions installed on the rightmost side of the levee foundation layer, it was found that the moving-average smoothing method was the most suitable analysis method. This technique is effective in understanding the levee seepage mechanism by comparing temperature changes at several positions, because the temperature data did not have any seasonality. However, it is suggested that the applicability of other data-processing techniques should also be examined in the future for simple monitoring or under other conditions and environments. Finally, by comparing the temperature changes measured at different heights and over time in the foundation layer, the positions inside the levees where seepage primarily occurred and its mechanism could be analyzed. 

For the practical implementation of the DTS system, further research considering long-term measurement results and various factors is necessary. Additionally, a comprehensive understanding of the external environment is essential for monitoring levee stability under seepage conditions using distributed optical-fiber sensors. As the temperature inside a levee varies depending on time and space, the entire area where stability needs to be ensured and local regions susceptible to damage must be monitored. The results of this study reconfirm that using an optical-fiber distributed temperature system with an appropriate post-treatment process can be an efficient monitoring method. 

## Figures and Tables

**Table 1:** Time when the water pressure change began and the time when there was little change in water pressure monitored by each sensor.

Levee 1			Levee 2		
Sensor Number	Distance [m]	Time [mm/dd hh:mm]	Sensor Number	Distance [m]	Time [mm/dd hh:mm]
G4	2 m	11/07 11:30 to 11/07 20:37	G10	2 m	11/07 11:41 to 11/07 20:44
G5	5 m	11/07 12:18 to 11/07 21:33	G11	5 m	11/07 12:42 to 11/07 21:43
G6	8 m	11/08 14:00 to 11/07 22:40	G12	8 m	11/08 14:18 to 11/08 22:35
G7	2 m	11/07 11:36 to 11/07 19:45	G13	2 m	11/07 11:31 to 11/07 20:30
G8	5 m	11/07 12:28 to 11/07 22:34	G14	5 m	11/07 12:36 to 11/07 22:54
G9	8 m	11/07 13:59 to 11/07 23:11	G15	8 m	11/07 13:55 to 11/07 23:34
G16	9 m	11/07 15:32 to 11/08 22:45	G17	9 m	11/07 15:21 to 11/08 23:39

## Data Availability

Not applicable.
